# Prescription Opioid Exposure During Pregnancy and Risk of Spontaneous Preterm Delivery

**DOI:** 10.1001/jamanetworkopen.2023.55990

**Published:** 2024-02-14

**Authors:** Olivia M. Bosworth, Maria C. Padilla-Azain, Margaret A. Adgent, Andrew J. Spieker, Andrew David Wiese, Amelie Pham, Ashley A. Leech, Carlos G. Grijalva, Sarah S. Osmundson

**Affiliations:** 1 Vanderbilt University, Nashville, Tennessee; 2 Department of Health Policy, Vanderbilt University Medical Center, Nashville, Tennessee; 3 Department of Biostatistics, Vanderbilt University Medical Center, Nashville, Tennessee; 4 Department of Obstetrics and Gynecology, Vanderbilt University Medical Center, Nashville, Tennessee

## Abstract

**Importance:**

Opioid exposure during pregnancy has been associated with preterm birth, but prior studies have not differentiated between spontaneous and indicated preterm birth or fully investigated these associations as functions of opioid dose.

**Objective:**

To determine whether prescription opioid use during pregnancy is associated with spontaneous preterm birth and whether the association is dose-dependent.

**Design, Setting, and Participants:**

This case-control study examined a retrospective cohort of pregnant patients enrolled in Tennessee Medicaid. Enrollment files were linked to health care encounters, hospital discharge information, birth certificate data, and prescription fills. Eligible participants were pregnant people ages 15 to 44 years without opioid use disorder who experienced birth of a single fetus at 24 weeks gestation or greater between 2007 and 2019 with linked birth certificate data. Cases of spontaneous preterm birth were matched with up to 10 controls based on pregnancy start date, race, ethnicity, age at delivery within 2 years, and history of prior preterm birth. Cases and matched controls were continuously enrolled in TennCare for at least 90 days prior to the index date (case delivery date).

**Exposure:**

Total opioid MME filled during the 60 days prior to the index date.

**Main Outcomes and Measures:**

The primary outcome was spontaneous preterm birth determined by a validated algorithm using birth certificate data. Conditional logistic regression was used to estimate the association between spontaneous preterm birth and total opioid morphine milligram equivalents (MME) dispensed, adjusting for parity, prepregnancy body mass index, education level, tobacco use, hepatitis infections, and pain indications.

**Results:**

A total of 25 391 cases (median [IQR] age, 23 [20-28] years; 127 Asian [0.5%], 9820 Black [38.7%], 664 Hispanic [2.6%]; 14 748 non-Hispanic White [58.1%]) with spontaneous preterm birth were identified and matched with 225 696 controls (median [IQR] age, 23 [20-27] years; 229 Asian [0.1%], 89 819 Black [39.8%], 3590 Hispanic [1.6%]; 132 002 non-Hispanic White [58.5%]) (251 087 patients total), with 18 702 patients (7.4%) filling an opioid prescription in the 60 days prior to the index date. Each doubling of nonzero opioid MME was associated with a 4% increase in the odds of spontaneous preterm birth compared with no opioid exposure (adjusted odds ratio, 1.04; 95% CI, 1.01-1.08).

**Conclusions and Relevance:**

In this case-control study, a positive association was found between total prescription opioid dose dispensed and the odds of spontaneous preterm birth. These findings support guidance to minimize opioid exposure during pregnancy and prescribe the lowest dose necessary.

## Key Points

**Question:**

What is the association between prescription opioid exposure during pregnancy and spontaneous preterm birth?

**Findings:**

In this case-control study of 251 087 pregnant patients with Tennessee Medicaid and without opioid use disorder, we found a continuous positive association between total opioid exposure and the odds of spontaneous preterm birth.

**Meaning:**

These findings support guidance to prescribe the lowest opioid dose necessary in pregnancy to manage pain.

## Teaser

The case-control study of pregnant patients in a US state Medicaid system examines the association of opioid exposure during pregnancy with spontaneous preterm birth.

## Introduction

Acute pain management during pregnancy is common for a broad range of etiologies, but clinicians have limited pharmacological treatments beyond acetaminophen for moderate to severe pain because nonsteroidal anti-inflammatory drugs have known fetal risks.^1,2^ Clinicians are left with opioid medications as the primary pharmacological treatment to treat pain not controlled with acetaminophen and this has likely contributed to high rates of opioids prescribed during pregnancy in the US.^3^ Some studies report that up to 21% of pregnant patients use opioids at some point in their pregnancy.^4^ Exposures such as medication-assisted therapy among women with opioid use disorders and outcomes such as neonatal opioid withdrawal syndrome have been well-studied.^5,6^ Nevertheless, the impact of a short prescription opioid exposure for acute episodes of pain on other perinatal outcomes is not well characterized.

Preterm birth is a major public health problem affecting up to 10.5% of pregnancies with profound consequences for infants and children, such as death, neurodevelopmental impairment, and chronic disease.^7^ Multiple studies have reported an association between opioid exposure during pregnancy and preterm birth, although the mechanism of this association is poorly defined.^5,8,9,10,11^ Some studies suggest this association is due to effects on placental development early in pregnancy, which leads to iatrogenic preterm birth due to maternal or fetal indications for delivery such as fetal growth restriction, placental abruption, or preeclampsia.^6^ Importantly, previous studies on prenatal opioid exposure have not distinguished between indicated and spontaneous preterm birth, which impacts clinicians’ ability to adequately counsel patients about the potential risks of opioid use, especially sporadic use, during pregnancy. Therefore, we sought to examine the association between short-term exposure to prescription opioids during pregnancy and spontaneous preterm birth and whether the association is dose-dependent.

## Methods

The study was approved by the institutional review board at Vanderbilt University Medical Center, the Tennessee Department of Health, and the Bureau of TennCare, who waived patient consent. This report followed the Strengthening the Reporting of Observational Studies in Epidemiology (STROBE) reporting guideline for case-control studies.

### Data Source

We performed a nested case-control study^12^ constructed from a retrospective cohort of pregnant patients enrolled in Tennessee Medicaid (TennCare), which provides insurance coverage to 50% of state pregnant patients.^13^ TennCare enrollment files were linked to health care encounters, hospital discharge data, vital records, and prescription fills. These files were also linked to Tennessee birth certificate data that provide information on clinical estimates of gestational age, maternal demographics and clinical characteristics, and obstetric procedures. Opioid pharmacy prescription data, including opioid type, tablet number, and strength, were identified using National Drug Codes.

### Study Cohort and Nested Case-Control Design

**Figure 1.**
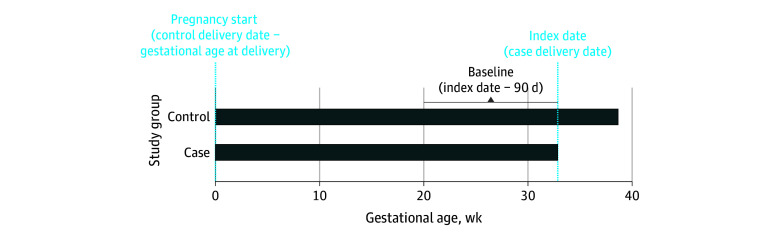
Study Design Overview of the case-control design of this study. Index date was date of delivery. Estimated gestational age at the time of delivery was used to calculate the pregnancy start date for both cases and controls. Cases were matched to controls whose pregnancy start date was within 4 days of the case’s start date and had not delivered prior to the index date. Controls could have a preterm delivery but at a date after the index dates.

The study cohort included pregnant people ages 15 to 44 years who experienced the birth of a single fetus at 24 weeks gestation or greater in a Tennessee hospital between 2007 and 2019 and whose records could be linked to a birth certificate. Within this retrospective cohort, cases of spontaneous preterm birth were identified using birth certificate data and the algorithm developed by Klebanoff et al^14^ and used by others^15,16,17^ where birth between 24 weeks 0 days and 36 weeks 6 days was considered likely spontaneous if there were premature rupture of membranes, prolonged or precipitous labor, a use or attempted use of forceps or a vacuum, and no induction for delivery. We used the estimated gestational age at the time of delivery to calculate the pregnancy start date for both cases and controls. The date of delivery for cases was the index date. Cases were matched to controls whose pregnancy start date was within 4 days of the case’s start date and had not delivered prior to the index date. Controls could have a preterm delivery but at a date after the index dates. Both cases and controls were required to have 90 days of continuous enrollment prior to the index date to enable ascertainment of exposure and covariates (Figure 1). These 90 days prior to the index date were considered the baseline period for both cases and matched controls. Patients missing data on race, ethnicity, maternal age, or history of preterm birth were excluded prior to case-control matching. In addition to the index date and pregnancy start date, each case was matched to up to 10 controls using incidence density sampling based on leading risk factors for preterm birth and included race, ethnicity, age at delivery within 2 years, and a history of prior preterm birth.^7,18^ Race and ethnicity were self-reported and were included due to racial disparities in the incidence of spontaneous preterm birth previously reported.^19^ Categories included Hispanic, non-Hispanic Asian, non-Hispanic Black, and non-Hispanic White. We also excluded patients with opioid use disorder based on *International Classification of Diseases, Ninth Revision *(*ICD-9*) and *International Statistical Classification of Diseases and Related Health Problems, Tenth Revision *(*ICD-10*) codes or filled prescriptions for buprenorphine during the baseline period. Buprenorphine was deemed to be a surrogate indicator of opioid use disorder. Finally, we excluded patients whose total opioid morphine milligram equivalent (MME) dose exceeded the 99.5th percentile (4200 MME) among patients filling prescriptions for opioids, as those rare scenarios likely represented implausible values or unique circumstances. Controls who were no longer matched to cases after the exclusions were applied were also excluded.

### Exposures

The primary exposure was total opioid MMEs filled during the 60 days prior to the index date and calculated from pharmacy data on opioid strength, type, and dispensed quantity using common conversion factors published by the Centers for Disease Control and Prevention.^20^ Liquid and patch formulations were included but we did not include opioids given in antitussive and antidiarrheal agents, which generally are prescribed for indications other than pain. We also classified the type of opioids filled in this period into 5 mutually exclusive categories (hydrocodone alone, oxycodone alone, codeine alone, other opioids alone, and 2 or more opioids).

### Covariates

Covariates were identified a priori based on potential associations with both opioid use during pregnancy and spontaneous preterm birth and included parity, prepregnancy body mass index (BMI), education level, tobacco use, hepatitis B and C infections, and pain indications. All covariates were derived from the birth certificate except pain indications, which were identified from administrative claims and hospital discharge data (*ICD-9, Clinical Modification *and *ICD-10, Clinical Modification*). Pain conditions included those associated with abdominal pain, myalgias, dental pain, trauma, chronic pain, malignant neoplasm, sickle cell disease, and autoimmune and inflammatory conditions.

### Statistical Analysis

Frequencies and medians with interquartile ranges were used to compare demographic and clinical characteristics between cases and controls. Descriptive comparisons for opioid dose were conducted by quartiles of total opioid MME. In total, 0.3% of covariate values were missing; to address this, we performed multiple imputation by chained equations with M = 25 iterations. We used conditional logistic regression for matched case-control data to estimate the association between spontaneous preterm birth and total opioid MME dispensed, adjusting for study covariates. We log transformed the total MME dose to address the high degree of right-skewness in the exposure and included an indicator term for a nonzero dose to reflect a possible spike-at-zero effect.^21^ Cluster robust standard errors were used to account for correlation among patients contributing multiple pregnancies to the cohort. In a secondary analysis, we also examined the association between spontaneous preterm birth and opioid type. A sensitivity analysis was performed to exclude deliveries for which participants filled opioid prescriptions three days prior to delivery because opioids can be prescribed for pain associated with delivery. All analyses were conducted using Stata BE version 18.0 (StataCorp) or R version 4.2.2 (R Project for Statistical Computing).

## Results

We identified 25 391 cases of spontaneous preterm birth (median [IQR] age, 23 [20-28] years; 127 Asian [0.5%], 9820 Black [38.7%], 664 Hispanic [2.6%]; 14 748 non-Hispanic White [58.1%]) and 225 696 matched controls (median [IQR] age, 23 [20-27] years; 229 Asian [0.1%], 89 819 Black [39.8%], 3590 Hispanic [1.6%]; 132 002 non-Hispanic White [58.5%]), for a total sample size of 251 087 from the retrospective cohort of eligible births (754 272 births) after applying exclusion criteria (the eFigure in Supplement 1). Cases were more likely to have completed only a high school education, had a lower BMI, and were more likely to be diagnosed with medical conditions associated with acute or chronic pain (Table 1). In aggregate, controls had a history of spontaneous preterm birth less frequently than cases. This is an artifact of cases with a history of spontaneous preterm birth matching to fewer number of controls (mean [SD], 3.53 [2.20]) than cases without a history of spontaneous preterm birth (mean [SD], 9.67 [1.44]), which has the effect of increasing the absolute number of controls without a history of spontaneous preterm birth and decreasing the frequency of this variable among controls. The distribution of opioid characteristics in case patients and matched control patients is presented in Table 2. In the 60 days prior to delivery, 8.8% of cases and 7.3% of controls filled an opioid prescription.

**Table 1.** Characteristics of Case Patients and Matched Control Patients^a^

Characteristics	Patients, No. (%)	
	Case (n = 25 391)	Control (n = 225 696)
Maternal age, median (IQR), y	23 (20-28)	23 (20-27)
Nulliparity		
Yes	8909 (35.3)	87 678 (39.0)
No	16 333 (64.3)	137 367 (60.9)
Missing	149 (0.6)	651 (0.3)
Previous preterm birth	2690 (10.6)	9162 (4.1)^b^
High school education or less	17 597 (69.6)	145 573 (64.7)
Missing	90 (0.4)	566 (0.3)
Prepregnancy BMI, median (IQR)	24 (21-30)	26 (22-31)
Missing	583 (2.3)	4655 (2.1)
Race and ethnicity^c^		
African American or Black	9820 (38.7)	89 819 (39.8)
Asian	127 (0.5)	229 (0.1)
Hispanic or Latinx	664 (2.6)	3590 (1.6)
Non-Hispanic White	14 748 (58.1)	132 002 (58.5)
Not categorized	32 (<0.1)	56 (<0.1)
Depression	1639 (6.5)	9044 (4.0)
Chronic hypertension	714 (2.8)	5116 (2.3)
Preexisting diabetes	478 (1.9)	2186 (1.0)
Pain conditions	4801 (18.9)	30 529 (13.5)
Abdominal	4503 (17.7)	29 158 (12.9)
Myalgia	161 (0.6)	1073 (0.5)
Dental	1 (<0.1)	21 (<0.1)
Trauma and accidents	56 (0.2)	456 (0.2)
Sickle cell disease	50 (0.2)	121 (0.1)
Malignant neoplasm	1 (<0.1)	0
Kidney stones	60 (0.2)	153 (0.1)
Inflammatory arthritis	33 (0.1)	66 (<0.1)
Colitis	46 (0.2)	118 (0.1)
Chronic pain	68 (0.3)	265 (0.1)
Acute pain	56 (0.2)	442 (0.2)

Abbreviation: BMI, body mass index (calculated as weight in kilograms divided by height in meters squared).

^a^ Cases and controls matched 1:N on maternal age, race, ethnicity, and history of preterm birth.

^b^ Imbalance due to patients with a history of spontaneous preterm birth matching to fewer number of controls than cases without a history of spontaneous preterm birth.

^c^ Race and ethnicity were self-reported.

**Table 2.** Distribution of Opioid Characteristics in Case Patients and Matched Control Patients^a^

Characteristics	Patients, No. (%)	
	Case (n = 25 391)	Control (n = 225 696)
Total opioid dose, MME		
0	23 164 (91.2)	209 221 (92.7)
1-74	489 (1.9)	4142 (1.8)
75-134	505 (2.0)	4038 (1.8)
135-278	583 (2.3)	4263 (1.9)
>278	650 (2.6)	4032 (1.8)
Opioid types^b^		
Hydrocodone	1079 (48.5)	8268 (50.2)
Codeine	510 (22.9)	4358 (26.5)
Oxycodone	233 (10.5)	1385 (8.4)
Propoxyphene	63 (2.8)	450 (2.7)
Tramadol	74 (3.3)	398 (2.4)
Meperidine	8 (0.4)	78 (0.5)
Hydromorphone	4 (0.2)	21 (0.1)
Morphine	5 (0.3)	11 (0.1)
Oxymorphone	1 (<0.1)	2 (<0.1)
Fentanyl	0	1 (<0.1)
Combination	250 (11.2)	1502 (9.1)

Abbreviation: MME, morphine milligram equivalents.

^a^ Cases and controls matched on maternal age, race, ethnicity, and history of preterm birth.

^b^ Denominator includes only patients who received opioids (2240 cases; 16 536 controls).

**Figure 2.**
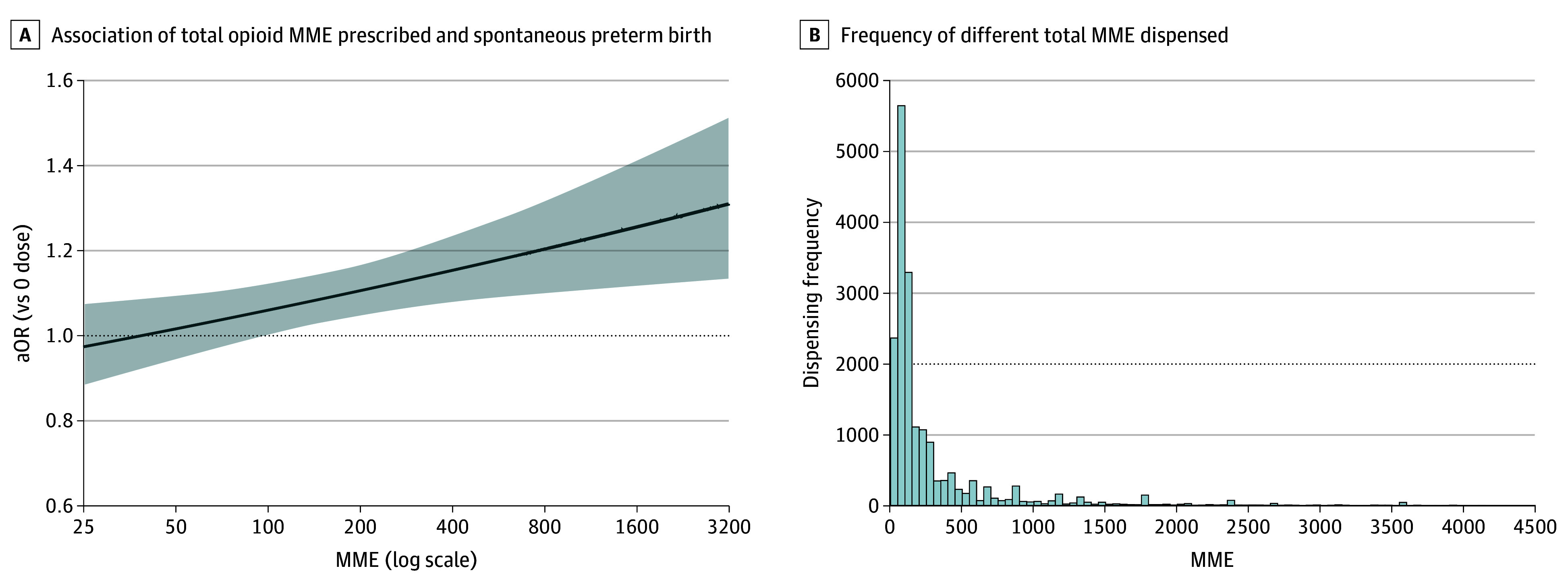
Total Opioid Morphine Milligram Equivalents (MME) Prescribed and Spontaneous Preterm Birth and Frequency of Different Total MME Dispensed In panel A, >98.7 MME is the point where the lower bound of the 95% CI becomes statistically significant. aOR indicates adjusted odds ratio.

Opioid MME dose prescribed in the 60 days prior to the index date was significantly associated with higher odds of spontaneous preterm birth as illustrated in Figure 2. Each doubling of nonzero opioid MME was associated with a 4% increase in the odds for spontaneous preterm birth compared with no opioid exposure (adjusted odds ratio [OR], 1.04; 95% CI, 1.01-1.08). Using this model, we estimated the odds for spontaneous preterm birth associated with common opioid MME dose prescriptions (Table 3). There were statistically significant higher odds for spontaneous preterm birth among individuals prescribed the most commonly filled opioid MME prescriptions, with evidence of significant increased odds of spontaneous preterm birth above 100 MME (Table 3; Figure 2). For example, a 150-MME opioid prescription would be associated with an 8% increase in the odds for spontaneous preterm birth compared to no opioid exposure (aOR, 1.08; 95% CI, 1.03-1.14). In contrast, a 450-MME opioid prescription would be associated with a 16% increased odds for spontaneous preterm birth compared with no opioid exposure (aOR, 1.16; 95% CI, 1.08-1.24). A total of 1573 pregnancies filled prescriptions for 900 MMEs or greater. A 900-MME opioid prescription would be associated with a 21% increased odds for spontaneous preterm birth compared with no opioid exposure (aOR, 1.21; 95% CI, 1.10-1.33). Compared with the lowest strength opioid, codeine, the estimated odds for spontaneous preterm birth did not substantially vary by opioid type after adjusting for confounders and opioid MME.

**Table 3.** Adjusted Logistic Regression Models of the Association of Opioid Dosage and Type With Spontaneous Preterm Birth

Characteristic	Patients, No.		Adjusted OR (95% CI)^a^
	Case^b^	Control	
Total MME (prescription)^c^			
0	23 164	209 221	1 [Reference]
50 (1 fill hydrocodone 5mg, 10 tablets)	98	804	1.01 (0.94-1.09)
150 (1 fill hydrocodone 5mg, 30 tablets)	155	1057	1.08 (1.03-1.14)
225 (1 fill oxycodone 5mg, 30 tablet)	66	436	1.11 (1.05-1.17)
300 (2 fills hydrocodone 5mg, 30 tablets)	57	379	1.13 (1.07-1.20)
450 (1 fill oxycodone 10mg, 30 tablet)	34	283	1.16 (1.08-1.24)
550 (2 fills oxycodone 5mg, 30 tablets)	5	26	1.17 (1.09-1.27)
900 (2 fills oxycodone 10mg, 30 tablets)	35	217	1.21 (1.10-1.33)
Opioid type^c^			
Codeine	510	4358	1 [Reference]
No opioids	23 164	209 221	1.01 (0.48-2.15)
Hydrocodone	1079	8268	1.05 (0.51-2.14)
Oxycodone	233	1385	1.27 (0.62-2.59)
Combination	250	1502	1.21 (0.60-2.44)
Other opioids^d^	155	962	1.29 (0.62-2.64)

Abbreviation: MME, morphine milligram equivalents.

^a^ Adjusted for parity, prepregnancy body mass index, high school education or less, tobacco use, hepatitis C, hepatitis B, and pain conditions.

^b^ Cases and controls matched on maternal age, race, ethnicity, and history of preterm birth.

^c^ Adjusted for total opioid MME in addition to the covariates for overall adjusted OR (parity, prepregnancy body mass index, high school education or less, tobacco use, hepatitis C, hepatitis B, and pain conditions).

^d^ Other opioids include dihydrocodeine, fentanyl, hydromorphone, meperidine, morphine, nalbuphine, oxymorphone, propoxyphene, tramadol, and butorphanol.

In our sensitivity analysis, we excluded opioid prescriptions dispensed within 3 days of the index date to account for possible opioid prescribing associated with pain from labor and potential protopathic issues. This reduced the number of deliveries with any opioid prescription filled by 655 (573 controls, 82 cases) but the findings of this analysis yielded results similar to those from the main analysis.

## Discussion

In this nested case-control study, we found a continuous association between total opioid MME exposure during midpregnancy and spontaneous preterm birth that remained unchanged after excluding opioids dispensed proximate to delivery for those with spontaneous preterm birth. We did not find any association between different opioid types after accounting for total opioid MME dispensed.

Each doubling of nonzero opioid MME was associated with a 4% increase in the odds for spontaneous preterm birth compared with no opioid exposure. This association may appear modest especially considering that common, 1-time prescriptions often fall in the 150 to 225 MME range, but these finding may provide more caution when prescribing multiple, higher strength opioids. In our cohort, 1573 pregnancies filled prescriptions for 900 MMEs or greater; 900 MME opioid prescriptions were associated with 21% increased odds for spontaneous preterm birth compared with no opioid exposure. We also caution against the conclusion that lower doses especially those below 100 MME are safe; the confidence bands over the low dose range still include odds ratios that are consistent with meaningful harm. More research would be required to determine confidently that lower dose opioid prescribing is not associated significantly with spontaneous preterm birth.

Because opioids cross the placenta and affect placental development, some investigators hypothesize that these adverse outcomes could be secondary to ischemic placenta disease from early pregnancy exposures that result in indicated preterm birth such as fetal growth restriction, abruption, and hypertensive disorders of pregnancy.^3^ Several studies have reported associations between opioid exposure during pregnancy and adverse pregnancy outcomes such as small for gestational age, placental abruption, and preterm birth.^5,8,9,10,22,23^ An association with preterm birth has been reported among those studies; however, it remains uncertain whether this association is causal or related to confounding exposures. Tobacco use is common among patients who used opioids and is also associated with preterm birth. Yet, accurately measuring tobacco use during pregnancy using claims data can be challenging. In a population-based study from Ontario limited to patients without opioid use disorder, the authors found that the association between opioid use and preterm birth was attenuated but not eliminated by addressing multiple potential sources of confounding.^5^ They also found that increasing cumulative opioid dose as estimated by the number of days supplied in pregnancy was associated with an increased risk of preterm birth.^5^ Other investigators have questioned whether this observed association could be due to the conditions leading to opioid use (ie, pain) rather than opioids themselves. Using a cohort from Sweden, where acetaminophen prescribing is regulated and can be tracked, Sujan et al^9^ found that comparing opioid-exposed pregnancies with acetaminophen-exposed pregnancies attenuated but did not eliminate associations between opioids and preterm birth.

### Strengths and Limitations

To our knowledge, no prior study directly addresses whether prenatal prescription opioid exposure is associated with spontaneous labor. A Pubmed search using the following terms yielded no relevant results: *((analgesics, opioid[MeSH Terms]) AND (preterm birth[MeSH Terms])) AND (spontaneous[Title/Abstract])*. Furthermore, our finding of a continuous association with opioid dose as measured by opioid MME and spontaneous preterm birth suggests a dose-response relationship. While a nested case-control study cannot prove this association, it does bolster the hypothesis of a causal relationship between opioid exposure and spontaneous preterm birth. We did not identify a statistically significant association between specific opioids and spontaneous preterm birth after accounting for opioid MME, suggesting that MME dose impacts spontaneous preterm birth more than opioid type.

Our study has several additional strengths that distinguish it from other studies. With detailed prescription data available, we were able to precisely quantify the amount of opioid dispensed during the exposure window lending to robust estimates of the association between opioid exposure and spontaneous preterm birth. While we did not define exposures by trimester as has been done in other studies, our study design ensured that all patients had exposures assessed during the same gestational age window during pregnancy minimizing the effect of pregnancy development and exposure timing on the outcome. We also enriched our data, using detailed information from birth certificates, including smoking, maternal weight, and other covariates that are often difficult to ascertain using other data sources. We limited our population to patients without opioid use disorder to reduce confounding and we also adjusted for multiple conditions related to pain to mitigate the impact of confounding by indication.

Nevertheless, this study is not without limitations. Despite a detailed and rich data source, we can only describe how many opioids were dispensed to patients, which is an imprecise proxy for actual use. However, this approach has been shown to be a reliable method for assessing medication exposures in claims data.^24,25^ We also do not have data on nonprescription analgesics used in pregnancy, which would help reduce concerns about residual confounding in our study. Although opioid use may be triggered by early initiation of labor and introduce potential protopathic concerns, our planned sensitivity analyses demonstrated that our findings were not affected by that potential phenomenon. We also acknowledge that we only examined births occurring at 24 weeks or greater, which limits our understanding of first trimester exposures and early pregnancy loss. In addition, our observations come from individuals enrolled in a Medicaid program, and our findings may not be generalizable to our populations or settings. Finally, we implemented a matched case-control study design in order to align the gestational age of exposure assessment for all comparisons, but residual confounding remains a concern. A case-control design requires that we report measures of association as an OR rather than relative risk, which is known to inflate the degree of association. Given that spontaneous preterm birth is a rare outcome (occurring in less than 10% of births), ORs generated from the analysis of case-control data approximate relative risks.^26^

## Conclusions

In summary, we found a continuous positive association between total prescription opioid MME dose exposure and the odds of spontaneous preterm birth. Our findings support guidance to prescribe the lowest dose necessary to manage pain.

## Notes

Supplement 1. **eFigure.** Structure of the Nested Case-Control Study

Supplement 2.
Data Sharing Statement

## References

[1] Li DK, Ferber JR, Odouli R, Quesenberry C. Use of nonsteroidal anti-inflammatory drugs during pregnancy and the risk of miscarriage. Am J Obstet Gynecol. 2018;219(3):275.e1-275.e8. doi:10.1016/j.ajog.2018.06.002 29890124

[2] US Food and Drug Administration. Nonsteroidal Anti-Inflammatory Drugs (NSAIDs): Drug Safety Communication—Avoid Use of NSAIDs in Pregnancy at 20 Weeks or Later. Last updated October 15, 2020. Accessed June 13, 2023. https://www.fda.gov/safety/medical-product-safety-information/nonsteroidal-anti-inflammatory-drugs-nsaids-drug-safety-communication-avoid-use-nsaids-pregnancy-20

[3] American College of Obstetricians and Gynecologists. Committee opinion No. 711: opioid use and opioid use disorder in pregnancy. Obstet Gynecol. 2017;130(2):e81-e94. doi:10.1097/AOG.0000000000002235 28742676

[4] Desai RJ, Hernandez-Diaz S, Bateman BT, Huybrechts KF. Increase in prescription opioid use during pregnancy among Medicaid-enrolled women. Obstet Gynecol. 2014;123(5):997-1002. doi:10.1097/AOG.0000000000000208 24785852 PMC4020039

[5] Brogly SB, Velez MP, Werler MM, Li W, Camden A, Guttmann A. Prenatal opioid analgesics and the risk of adverse birth outcomes. Epidemiology. 2021;32(3):448-456. doi:10.1097/EDE.0000000000001328 33625160 PMC8011506

[6] Esposito DB, Huybrechts KF, Werler MM, . Characteristics of prescription opioid analgesics in pregnancy and risk of neonatal opioid withdrawal syndrome in newborns. JAMA Netw Open. 2022;5(8):e2228588. doi:10.1001/jamanetworkopen.2022.28588 36001312 PMC9403776

[7] American College of Obstetricians and Gynecologists. Prediction and prevention of spontaneous preterm birth: ACOG practice bulletin, number 234. Obstet Gynecol. 2021;138(2):e65-e90. doi:10.1097/AOG.0000000000004479 34293771

[8] Interrante JD, Scroggs SLP, Hogue CJ, ; National Birth Defects Prevention Study. Prescription opioid use during pregnancy and risk for preterm birth or term low birthweight. J Opioid Manag. 2021;17(3):215-225. doi:10.5055/jom.2021.0632 34259333 PMC8637424

[9] Sujan AC, Quinn PD, Rickert ME, . Maternal prescribed opioid analgesic use during pregnancy and associations with adverse birth outcomes: a population-based study. PLoS Med. 2019;16(12):e1002980. doi:10.1371/journal.pmed.1002980 31790390 PMC6886755

[10] Graeve R, Balalian AA, Richter M, . Infants’ prenatal exposure to opioids and the association with birth outcomes: a systematic review and meta-analysis. Paediatr Perinat Epidemiol. 2022;36(1):125-143. doi:10.1111/ppe.12805 34755358

[11] Flannagan KS, Sjaarda LA, Mumford SL, Schisterman EF. Prescription opioid use among populations of reproductive age: effects on fertility, pregnancy loss, and pregnancy complications. Epidemiol Rev. 2020;42(1):117-133. doi:10.1093/epirev/mxaa007 33001215

[12] Wiese AD, Griffin MR, Schaffner W, . Opioid analgesic use and risk for invasive pneumococcal diseases: a nested case-control study. Ann Intern Med. 2018;168(6):396-404. doi:10.7326/M17-1907 29435555 PMC6647022

[13] Division of TennCare. TennCare Overview. Accessed June 13, 2023. https://www.tn.gov/tenncare/information-statistics/tenncare-overview.html

[14] Klebanoff MA, Yossef-Salameh L, Latimer C, . Development and validation of an algorithm to determine spontaneous versus provider-initiated preterm birth in US vital records. Paediatr Perinat Epidemiol. 2016;30(2):134-140. doi:10.1111/ppe.12267 26860444

[15] Stout MJ, Demaree D, Merfeld E, . Neonatal outcomes differ after spontaneous and indicated preterm birth. Am J Perinatol. 2018;35(5):494-502. doi:10.1055/s-0037-1608804 29183099 PMC10507481

[16] Stout MJ, Macones GA, Tuuli MG. Accuracy of birth certificate data for classifying preterm birth. Paediatr Perinat Epidemiol. 2017;31(3):245-249. doi:10.1111/ppe.12352 28370345

[17] Ananth CV, Joseph KS, Oyelese Y, Demissie K, Vintzileos AM. Trends in preterm birth and perinatal mortality among singletons: United States, 1989 through 2000. Obstet Gynecol. 2005;105(5 Pt 1):1084-1091. doi:10.1097/01.AOG.0000158124.96300.c7 15863548

[18] Esplin MS, O’Brien E, Fraser A, . Estimating recurrence of spontaneous preterm delivery. Obstet Gynecol. 2008;112(3):516-523. doi:10.1097/AOG.0b013e318184181a 18757647

[19] Osterman MJK, Hamilton BE, Martin JA, Driscoll AK, Valenzuela CP. Births: final data for 2021. Natl Vital Stat Syst. 2023;72(1):1-53. 36723449

[20] Dowell D, Ragan KR, Jones CM, Baldwin GT, Chou R. CDC clinical practice guideline for prescribing opioids for pain—United States, 2022. MMWR Recomm Rep. 2022;71(3):1-95. doi:10.15585/mmwr.rr7103a1 36327391 PMC9639433

[21] Lorenz E, Jenkner C, Sauerbrei W, Becher H. Modeling variables with a spike at zero: examples and practical recommendations. Am J Epidemiol. 2017;185(8):650-660. doi:10.1093/aje/kww122 28369154

[22] Corsi DJ, Hsu H, Fell DB, Wen SW, Walker M. Association of maternal opioid use in pregnancy with adverse perinatal outcomes in Ontario, Canada, from 2012 to 2018. JAMA Netw Open. 2020;3(7):e208256. doi:10.1001/jamanetworkopen.2020.8256 32725246 PMC12064095

[23] Wen X, Wang S, Lewkowitz AK, Ward KE, Brousseau EC, Meador KJ. Maternal complications and prescription opioid exposure during pregnancy: using marginal structural models. Drug Saf. 2021;44(12):1297-1309. doi:10.1007/s40264-021-01115-6 34609720 PMC8830421

[24] Johnson RE, Vollmer WM. Comparing sources of drug data about the elderly. J Am Geriatr Soc. 1991;39(11):1079-1084. doi:10.1111/j.1532-5415.1991.tb02872.x 1753045

[25] Ray WA, Griffin MR. Use of Medicaid data for pharmacoepidemiology. Am J Epidemiol. 1989;129(4):837-849. doi:10.1093/oxfordjournals.aje.a115198 2646920

[26] Norton EC, Dowd BE, Maciejewski ML. Odds ratios—current best practice and use. JAMA. 2018;320(1):84-85. doi:10.1001/jama.2018.6971 29971384

