# Supplementary material for: Prescription Opioid Exposure During Pregnancy and Risk of Spontaneous Preterm Delivery
Source: JAMA Netw Open. 2024 Feb 14;7(2):e2355990. doi: 10.1001/jamanetworkopen.2023.55990 (PMC10867678; doi:10.1001/jamanetworkopen.2023.55990)
Supplement: Supplement 1. — eFigure. Structure of the Nested Case-Control Study [file jamanetwopen-e2355990-s001.pdf]

Supplemental Online Content

Bosworth OM, Padilla-Azain MC, Adgent MA, et al. Prescription opioid exposure during pregnancy and risk of spontaneous preterm delivery. *JAMA Netw Open.* 2024;7(2):e2355990. doi:10.1001/jamanetworkopen.2023.55990

This supplemental material has been provided by the authors to give readers additional information about their work.

© 2024 Bosworth OM et al. *JAMA Network Open.*

eFigure 1: Structure of the nested case-control study

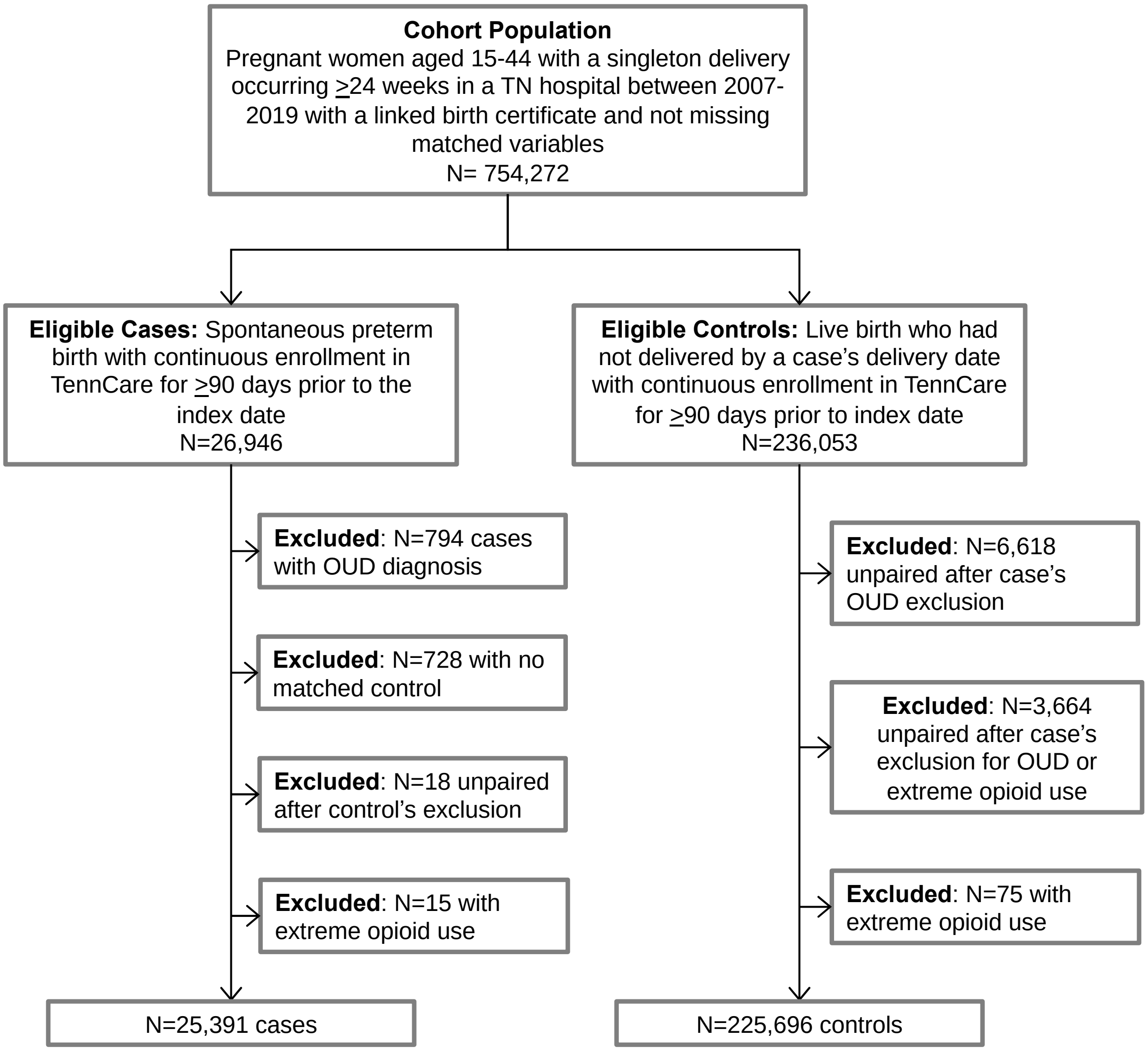

© 2024 Bosworth OM et al. *JAMA Network Open.*
