# Supplementary material for: Prescription Opioid Exposure During Pregnancy and Risk of Spontaneous Preterm Delivery
Source: JAMA Netw Open. 2024 Feb 14;7(2):e2355990. doi: 10.1001/jamanetworkopen.2023.55990 (PMC10867678; doi:10.1001/jamanetworkopen.2023.55990)
Supplement: Supplement 2. — Data Sharing Statement [file jamanetwopen-e2355990-s002.pdf]

Data Sharing Statement

Bosworth. Prescription Opioid Exposure During Pregnancy and Risk of Spontaneous Preterm Delivery. *JAMA Netw Open*. Published February 14, 2024.
doi:10.1001/jamanetworkopen.2023.55990

Data
Data available: No

Additional Information
Explanation for why data not available: Per data use agreement with the Tennessee Department of Health, we cannot share data
